# Associations of NF-kappaB and Bax with Apoptosis in Varicose Veins of Women of Different Age Groups

**DOI:** 10.1155/2011/639720

**Published:** 2011-11-01

**Authors:** Helle Evi Simovart, Andres Arend, Jüri Lieberg, Marina Aunapuu

**Affiliations:** ^1^Department of Anatomy, Biomedicum, University of Tartu, 19 Ravila Street, 50411 Tartu, Estonia; ^2^Surgery Clinic, University of Tartu, 8 L. Puusepa Street, 51014 Tartu, Estonia; ^3^Department of Morphology, Estonian University of Life Sciences, 62. F. Kreutzwaldi Street, 51014 Tartu, Estonia

## Abstract

The study aimed at detecting apoptotic endothelial cells (ECs) and smooth muscle cells (SMCs) together with determining expression of NF-kappaB (p105/p50) and Bax in varicose vein walls. Women (*n* = 35) undergoing the excision of varicose veins were divided into 3 groups: younger than 35 years (I), 36–50 years (II), and older than 50 years (III). Apoptosis was determined by the TUNEL method, NF-kappaB and Bax expression by immunohistochemistry. The percentage of apoptotic ECs and SMCs in the layers of varicose vein wall increased in groups II and III. NF-kappaB expression had the lowest level in Group II with particularly low level in the media. Contrariwise, Bax expression levels in Group II were increased. The study revealed that in varicose veins ECs and SMCs apoptosis increased with advancing age. If increase in apoptosis during earlier stages of varicosities is probably regulated by intrinsic pathway, then in older patients other signaling pathways may be involved.

## 1. Introduction

Varicose veins of legs are one of the commonest diseases affecting people worldwide. However, despite extensive studies, the etiology of varicose veins is still unclear as well as molecular mechanisms that could potentially lead to the dilation of the vein wall. Defects in the cellular and extracellular matrix composition of the vein wall are believed to cause weakness and alter venous tone, resulting in the formation of varicose veins [1]. Dysfunctional endothelial cells (ECs) and smooth muscle cells (SMCs) are probably the crucial regulators in the cascade of events leading to the restructuring of the vein wall with consequent venous dilatation. The endothelium regulates venous tone through release of vasoactive substances constricting and relaxing the veins. Both reduction of factors contributing to vasoconstriction and upregulation of factors contributing to vasodilatation have been shown in varicose veins [2–4]. Previously we have also shown that ECs of varicose veins appear desquamated and degenerated under electron microscopy [5]. As reported by Wali and Eid [6, 7], damaged ECs stimulate the recruitment of leukocytes and the release of growth factors, causing SMC hypertrophy, proliferation, and migration into the intima. Hypertrophy of SMCs in the media of varicose veins has also been described by Lee et al. [8]. One of the factors which can alter vein wall structure is dysregulated apoptosis. This may cause dedifferentiation of SMCs, transforming them from a contractile to a secretory type leading to the irregular fibrotic nature of the varicose vein wall [9]. Concerning the role of SMC apoptosis in varicose veins, controversial data have been obtained. Several authors [10–12] have reported reduction of the number of apoptotic SMCs in varicose veins compared with nonvaricose veins. Contrariwise, Bujan and co-authors [13] have found an increase of apoptotic SMCs in varicose veins. Urbanek and coauthors [14] have described an increase of the apoptotic index in the media of varicose veins of patients younger than 50 years, but not in the older age group. On the other hand, much less attention has been paid to the apoptotic regulation of endothelial cells in varicose veins. 

Two regulatory pathways of apoptosis are recognised. The intrinsic pathway (mitochondria pathway) and the extrinsic pathway (transmembrane pathway) are induced by various cellular and external stimuli and are regulated by the activation of different caspases [15]. The intrinsic pathway of apoptosis occurs as a consequence of mitochondrial permeabilization and is regulated by proapoptotic proteins such as Bcl-2-associated protein x (Bax), and it involves specific caspases, especially caspase 9 [16]. Bax is a protein that forms a homodimer or heterodimers with Bcl-2, and apoptosis depends on the ratio of these two proteins because it is promoted by Bax and inhibited by Bax/Bcl-2 heterodimers [17]. The extrinsic pathway is regulated by proteins such as Fas (also called Apo-1 or CD95), and it involves specific caspases, in particular caspase 8, a connecting ligand binding at the cell surface to apoptotic induction [18].

Apoptotic dysregulation in varicose veins is shown to be mediated via the intrinsic pathway. Ducasse and coauthors [10] have immunohistochemically quantified fewer Bax, and caspase-9-positive cells in the wall of varicose veins compared to healthy veins, but no changes were detected in Fas and caspase 8 immunoreactivity. Lee with co-authors [8, 19] have found dysregulation of apoptosis through the intrinsic pathway in the varicose veins and in the internal spermatic veins of patients with varicosele, which allows the authors to speculate that the mechanism of reduced apoptosis might contribute to the dilated and thickened walls of both venous diseases.

Expression levels of apoptotic proteins have been reported to be upregulated by the nuclear transcription factor kappa B (NF-kappaB) which, beside being a central regulator of the innate and adaptive immune response, is commonly described as an antiapoptotic transcription factor [16, 17], although under certain circumstances NF-kappaB might also positively contribute to apoptosis induction [20–22]. Many common diseases (cancer, atherosclerosis, diabetes) are also associated with the dysregulation of NF-kappaB signaling pathways that control its activity. Dysregulation of apoptosis, dependent on NF-kappaB activity, may also play an important role in the development of varicosities.

In our study we focused on the changes of the expression of molecular mediators NF-kappaB and Bax in the different layers of varicose vein walls in women of different age groups and attempted to associate it with the changes in the number of apoptotic ECs and SMCs. 

## 2. Materials and Methods

### 2.1. Patients and Samples

Samples from great saphenous veins of 35 female patients undergoing surgery for varicosities were studied. The patients were divided into groups according to their age: Group I (younger than 35 years), Group II (36–50 years), and Group III (older than 50 years). More details about the groups and patients are given in Table 1.

The protocol for the research project has been approved by the Ethics Review Committee on the Human Research of the University of Tartu, and it is in accordance with the Declaration of Helsinki (1964). Informed consent was obtained from each patient. 

The patients were operated at the Surgery Clinic, the University of Tartu, Estonia.

Patients with any concomitant disease were excluded from the study. Among patients investigated by us, there were three women with overweight and one woman who smoked. In our patients hormonal replacement therapy was not recorded, but 70% of the patients reported varicose veins in their parents.

Vein specimens were fixed in 10% buffered formalin, embedded in paraffin, and cut into sections of four micrometer thickness.

### 2.2. Detection of Apoptosis

The paraffin sections were dewaxed and rehydrated, and the in situ cell death detection kit, Fluorescein (Roche Diagnostics GmbH, Mannheim, Germany) was used for the detection of apoptotic cells according to the manufacturer's protocol as described earlier [23, 24]. The sections were permeabilized with proteinase K, PCR grade (Roche Diagnostics GmbH, Mannheim, Germany) and washed with phosphate-buffered saline (PBS), pH 7.4. The apoptotic cells were determined by terminal deoxynucleotidyl transferase-(TdT-) mediated deoxyuridinetriphosphate-(dUTP-) fluorescein nick end labelling of the free 3′-OH DNA terminal of fragmented DNA present in the apoptotic cells (TUNEL). For negative controls sections were incubated with solution without terminal transferase.

Apoptotic nuclei appeared to be green, whereas nuclei without strand breaks were not labelled. The apoptotic cells were counted with the microscope Zeiss Axiophot 2 in UV light, using a ×40 objective. Apoptotic index was calculated as percentage of TUNEL-positive cells by the following equation: apoptotic cells % = (number of TUNEL-positive cells/total number of cells) × 100.

### 2.3. Immunohistochemical Investigations

The localisation of NF-kappaB and Bax was determined in the endothelium, the subendothelial layer, the media, and the adventitia immunohistochemically, using the NF-kappaB p105/p50 antibody (ab7971, Abcam, Cambridge, UK) and the rabbit polyclonal antibody to Bax (ab16837, Abcam, Cambridge, UK). The staining procedure was continued with the Vectastain R Universal Elite ABC Kit (Vector Laboratories, Burlingame, Calif USA). In brief, the protocol combined the following steps. Four-*μ*m-thick paraffin sections were dewaxed, rehydrated, and washed for 5 minutes in PBS, pH 7.4. Endogenous peroxidase activity was blocked by 3% hydrogen peroxide (H_2_O_2_) solution incubating the sections for 30 minutes in a humidified chamber, then washed with PBS and incubated with a diluted normal blocking serum prepared from the same species in which the secondary antibody was produced. After that the sections were incubated with the primary antibody in dilutions 1 : 30 (NF-kappaB) and 1 : 50 (Bax) overnight. The sections were washed with PBS and incubated with a diluted biotinylated secondary antibody solution for 45 minutes at room temperature. Then the sections were rinsed with PBS and incubated for 30 minutes at room temperature with Vectastain^®^ Elite ABC Reagent and with 3,3′-diaminobenzidine (DAB)+Chromogen (Dako Cytomation, Carpinteria, Calif, USA) for 10–15 minutes and counterstained with Harris' haematoxylin. Immunostaining negative controls were performed by omitting primary antibody.

The intensities of the expressions of NF-kappaB and Bax were estimated visually with a light microscope using ×40 objective, and the staining intensity was graded from + (minimum) to ++++ (maximum) by two independent observers in a blind fashion. If the results of the two observers were not matching, the discordant cases were reevaluated by the two observers together. 

### 2.4. Statistical Analysis

The data were analyzed and presented as mean ± SEM. Statistical analyses were conducted by the ANOVA (count of apoptotic endothelial and smooth muscle cells) and the Mann-Whitney *U* test (evaluation of the immunohistochemical staining of Bax and NF-kappaB). Statistical significance was accepted when *P* < 0.05. 

## 3. Results

**Figure 1:**
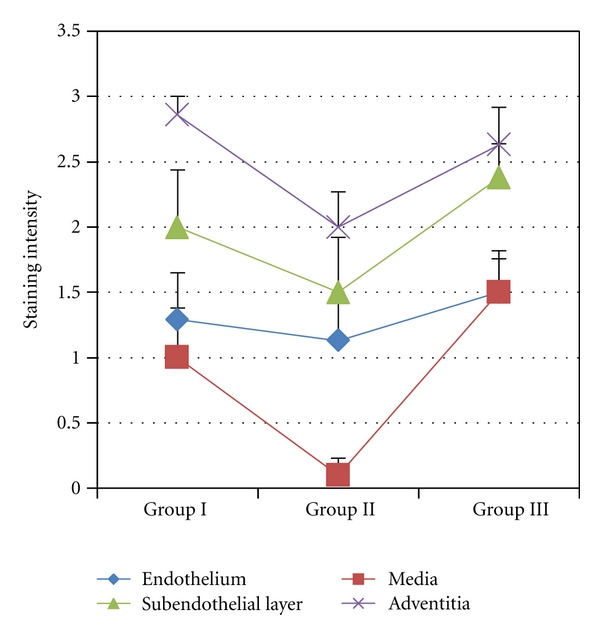
Changes of NF-kappaB expression in the wall of varicose veins. Statistically significant differences in the media—Group II versus Group III *P* < 0.05, in the adventitia—Group I versus Group II *P* < 0.05.

**Figure 2:**
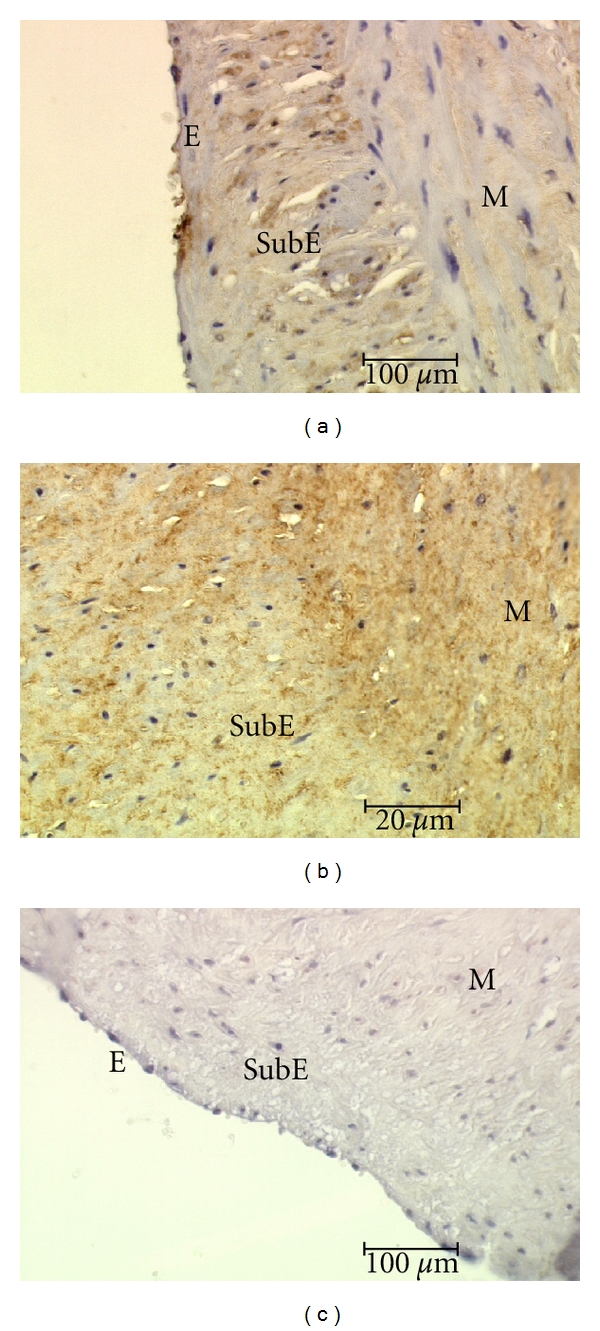
Expression of the NF-kappaB in the endothelium (E), subendothelial layer (SubE), and media (M). Group II (a) and Group III (b). (c) represents negative control (Group II).

Results of the evaluation of NF-kappaB (p105/p50) immunostaining intensity in the walls of varicose veins of women of different age groups are summarized in Figure 1. Our investigations showed that the p105/p50 expression was at the lowest level in Group II in all layers of the vein wall as compared to Groups I and III. When comparing layers of the vein wall, the highest p105/p50 expression was seen in the adventitia and the lowest in the media (Figure 1). Particularly in Group II the expression of p105/p50 in the media was absent in some cases or had a low grade (Figure 2(a)). On the contrary, in Groups I and III p105/p50 levels were higher in all layers of the vein wall (Figure 2(b)).

**Figure 3:**
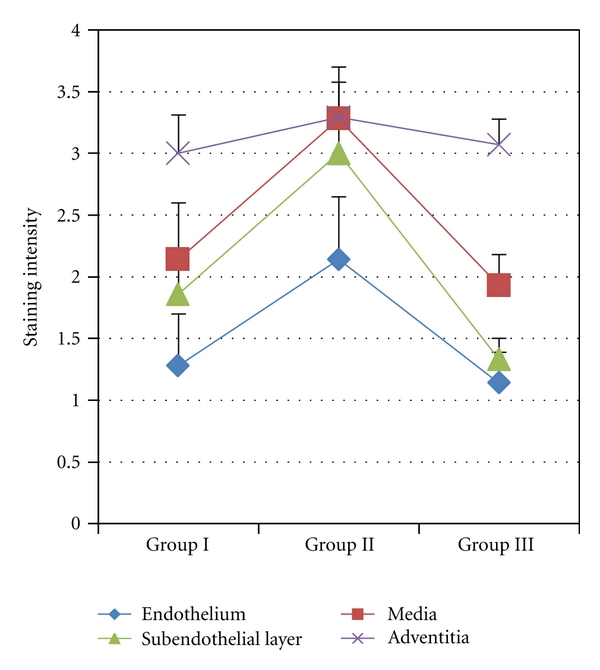
Changes of Bax expression in the walls of varicose veins. Statistically significant differences in the endothelium; Group II versus Group III *P* < 0.01, in the subendothelial layer; Group I versus Group II *P* < 0.05 and Group II versus Group III *P* < 0.05, in the media-Group I versus Group II *P* < 0.01 and Group II versus Group III *P* < 0.05.

**Figure 4:**
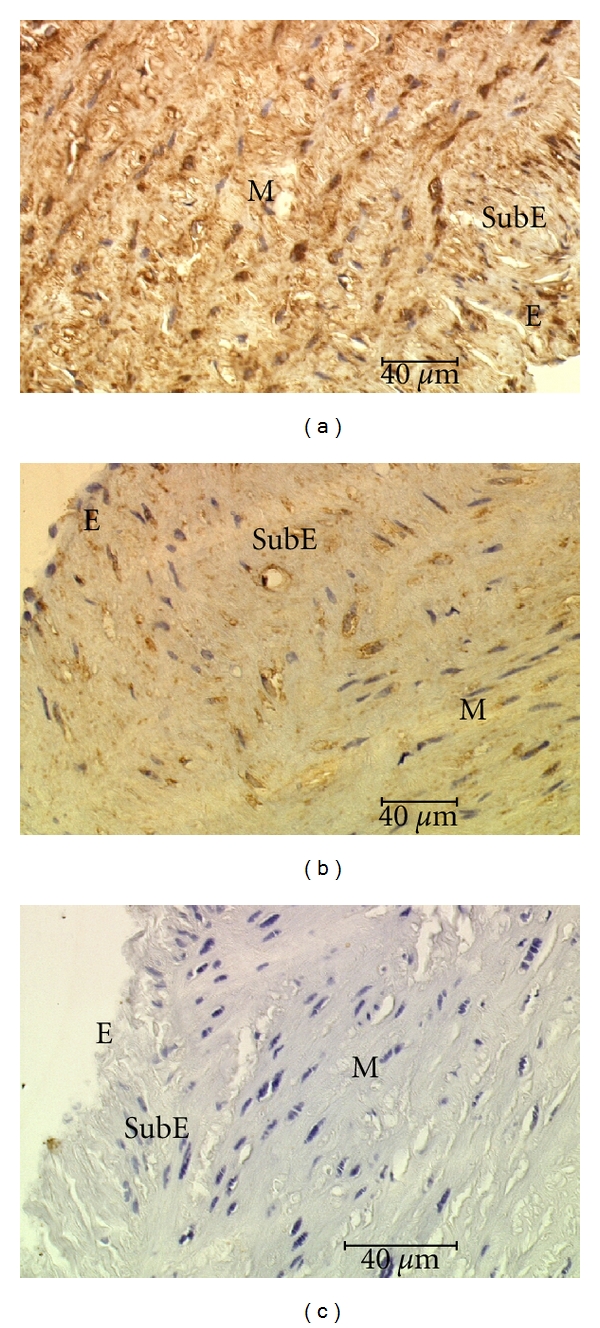
Expression of the Bax in the endothelium (E), subendothelial layer (SubE), and media (M). Group II (a) and Group III (b). (c) represents negative control (Group III).

Bax positive immunostaining appeared in the cells of all layers of varicose veins and in Group II a strong staining of Bax was seen in the adventitia, the media and the subendothelial layer, while in the endothelium staining was moderate (Figure 3). The most intensive Bax staining was found in the media in Group II (Figure 4(a)). In Group I and Group III Bax staining intensity was equally lower in all layers of the vein wall (Figure 4(b)) as compared to Group II, only expression of Bax in the adventitia stayed almost at the same level in all groups (Figure 3).

**Figure 5:**
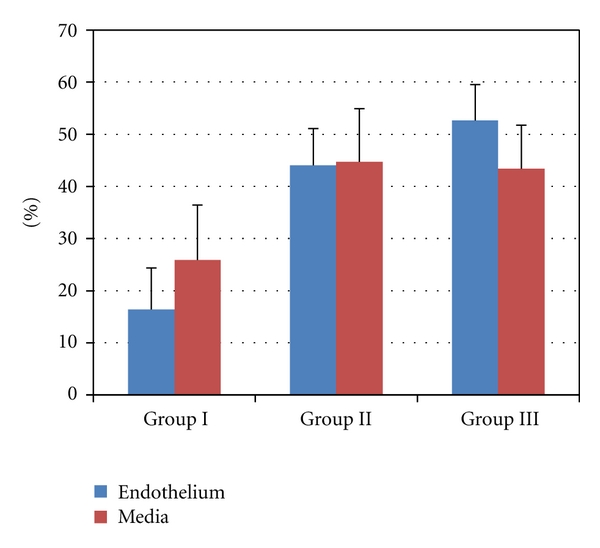
Changes in the percentage of apoptotic endothelial cells and smooth muscle cells. Statistically significant differences in the percentage of apoptotic endothelial cells were between Group I versus Group II *P* < 0.05 and Group I versus Group III *P* < 0.01.

**Figure 6:**
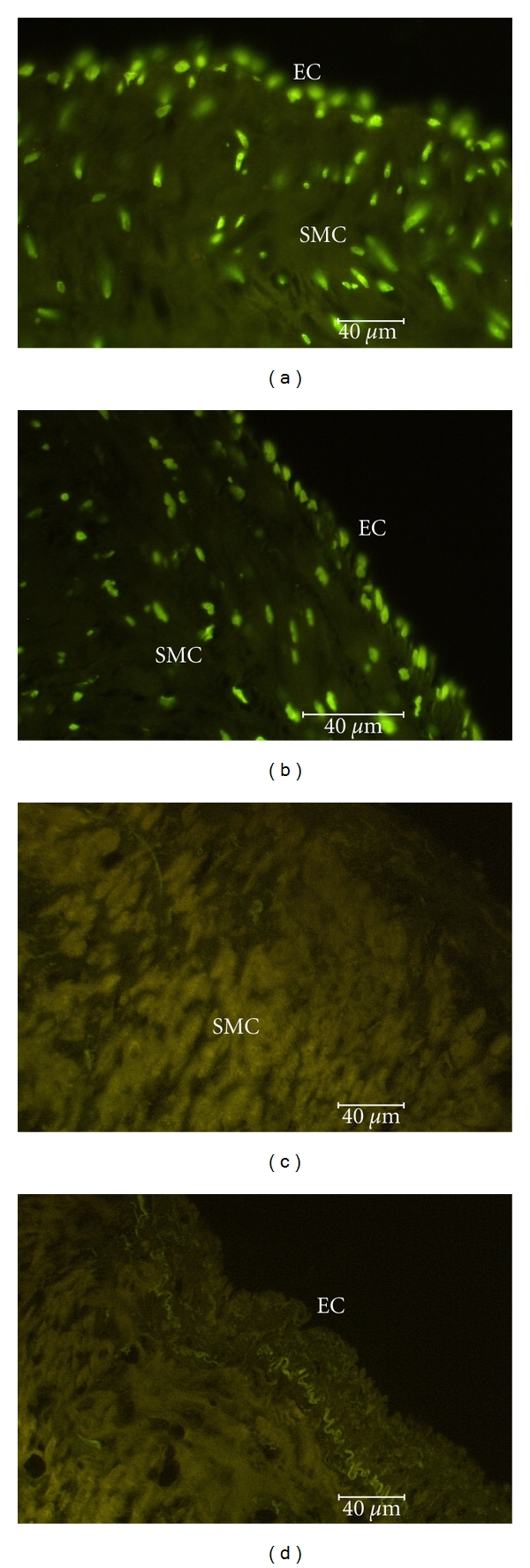
Apoptotic smooth muscle cells (SMCs), and endothelial cells (ECs). Group II (a) and Group III (b). (c) and (d) represent negative controls (Group II).

The count of apoptotic EC and SMC as detected by the TUNEL method in varicose veins increased in Groups II and III with the advancing age of patients. As shown in Figure 5, in Group II and especially in Group III the percentage of apoptotic EC and SMC was increased. Representative pictures of apoptotic SMC and EC are seen in Figure 6. In Group III an increase of apoptotic cells was also seen in vasa vasorum.

## 4. Discussion

In this study, as in our earlier investigation [24], we found a trend of the number of apoptotic cells to increase in the walls of varicose veins along with the advancing age. An increase of apoptotic SMCs and, in particular, apoptotic ECs was seen in older age groups (Figure 5). Still, the advancing age can itself be a factor that sensitizes cells to apoptosis. When assaying human umbilical vein endothelial cells, Wagner and coauthors [25] have shown an age-related increase in apoptosis, while Hoffmann and coauthors [26] demonstrated that aging increased the sensitivity of cells to apoptosis. Thus, in the group of older patients of our study, the increased number of apoptotic cells seen in the wall of varicose veins may be the result of two processes, an increased sensitivity to apoptosis induced by aging and the long-lasting course of the disease leading the pathology to a more advanced stage. As concerns aging, there are few studies on apoptosis in the varicose vein walls in which different age groups of patients have been compared. Urbanek and co-authors [14] have reported an increase of apoptotic SMCs in the varicose veins of subjects under 50 years of age, but not in older patients, which circumstance leads them to the speculation that apoptosis plays a role in the initial stage of the pathology but not in the advanced stages of the disease. Nevertheless, dysregulated apoptosis in the walls of varicose veins is attributed to the pathogenesis of the disease [1]. If Bujan and co-authors [13] have found an increase of apoptotic SMCs in the varicose vein wall, then others [10–12, 14] have reported a reduced number of apoptotic cells in varicose veins. Our preliminary data also allow us to speculate that the apoptosis of SMCs in non-varicose veins is higher than that in varicose veins, while in the case of apoptotic ECs the situation is likely the opposite; that is, apoptosis of ECs in nonvaricose veins may be lower. One of the pathogenetic mechanisms of dysregulated SMC apoptosis in the varicose vein wall may be connected with SMCs dedifferentiation from a contractile phenotype to a secretory one. Several investigations [27–29] have found that fibrotic degeneration with the disruption of the elastic network seen in varicose veins may be related to the accumulation of secretory SMCs. Increased ECs apoptosis seen in older age groups of our investigation may result in a partial loss of endothelial lining that we have also described in our previous studies [5]. Applying transmission electron microscopy, degeneration and a loss of ECs in varicose veins have also been reported by Wali and Eid [6]. Impairment of the endothelial layer may in turn activate signaling cascades that induce SMC migration to the subintima and cause their proliferation and a synthesis of large quantities of the extracellular matrix [30, 31].

In apoptotic signaling in varicose veins, the intrinsic but not the extrinsic pathway has been found to be involved. Both Ducasse with co-authors [10] and Lee with co-authors [8] have reported the downregulation of proapoptotic proteins Bax or Bcl-2 and caspase 9 in the walls of varicose veins showing the involvement of the intrinsic pathway, while no changes were detected in Fas and caspase 8 characterizing the extrinsic signaling pathway. Also Ascher and co-authors [12] have shown that the entry of SMCs into the apoptotic pathway is regulated by Bax, which is downregulated in varicose veins in comparison with healthy veins. In our study we were also able to follow changes in the Bax expression in the layers of varicose veins of different age groups. An increase in the Bax expression in Group II (ages of patients between 36 and 50 years) was found in the intima (including the endothelium), in the media, but not in the adventitia as compared to both younger and older patient groups, that is, Groups I and III (Figure 3). These results support the hypothesis that there may be a gradual increase of apoptosis during the earlier stages of the pathology, while in advanced stages the role of apoptosis may be less important. This speculative mechanism is supposed to exist by Urbanek and co-authors [14], who have found an increased apoptotic index of the media as well as a higher Bax expression in the varicose veins of patients younger than 50 years, but not in older subjects. Relatively higher EC and SMC apoptosis and lower levels of Bax in the intima and media of varicose veins of patients over 50 years of age (Group III) documented in our study may be explained by shifts in the sensitivity of cells to apoptosis caused by aging or by changed signaling during the advanced stage of the pathology; for example, instead of the intrinsic pathway, the extrinsic apoptotic signaling cascade is executed. Furthermore, the apoptosis of ECs and SMCs in the walls of varicose veins may be differently regulated or these cells react differently to apoptotic stimuli. Sata and co-authors [32] have reported a different sensitivity of ECs and SMCs to Fas-mediated apoptosis. In their in vitro experiment after stimulation with tumor necrosis factor *α* (TNF-*α*) or interferon-gamma (IFN-*γ*), the upregulation of Fas was seen in ECs but not in vascular SMCs. Similarly, in our study of elderly patients in Group III, the apoptosis of ECs tends to increase, while the apoptosis of SMCs remained on the same level as in Group II (Figure 5).

When comparing the expression of NF-kappaB (p105/p50) in the walls of varicose veins of patients of three age groups of our study, the lowest levels were noted in Group II. Low NF-kappaB expression was seen in all layers; it was the most remarkable in the media and less obvious in the endothelium, thus, again supporting the notion of differential regulation of SMCs and ECs. Both in Groups I and III higher NF-kappaB levels were found in the varicose vein wall (Figure 1). As seen from our investigations, a decrease in the NF-kappaB level in Group II was associated with an increase in the Bax expression in the same group (Figures 1 and 3). NF-kappaB, beside its pivotal roles in immune and inflammatory responses, is considered to be a critical player in the control of the apoptotic response to a variety of stimuli [21]. NF-kappaB is most commonly involved in the suppression of apoptosis by transactivating the expression of antiapoptotic genes. It has also been shown that NF-kappaB can inhibit the expression of Bax. Bentires-Alj and co-authors [33] have demonstrated that NF-kappaB regulates the Bax gene expression through the indirect pathway. In our study, when comparing groups of young and older patients, that is, Group I versus Group II, an increase in the apoptotic EC and SMC count was noticed in Group II together with a higher level of proapoptotic Bax and a reduced level of antiapoptotic NF-kappaB (p105/p50). The described changes were particularly evident in the medial layer of varicose veins. In patients of Group III the apoptosis of EC and SMC stayed high, though in the vein wall the expression of Bax decreased and p105/p50 increased to the level seen in Group I. This discrepancy can be explained by the process of aging, which may sensitize cells to apoptosis, with other signaling cascades activated. It can also be speculated that the p105/p50 increase in the varicose veins of older patients of Group III with an advanced stage of the disease may be related to the augmentation of the inflammatory component in the walls of varicose veins as the NF-kappaB signaling cascade may be activated both by inflammation and the aging process [34, 35].

## 5. Conclusion

In conclusion, our study demonstrated that in varicose veins the apoptosis of ECs and SMCs increases with the advancing age. When comparing the first two age groups of patients with presumably earlier stages of the pathology, increasing apoptotic activity can be associated with a higher level of Bax and a lower level of NF-kappaB (p105/p50). This process was seen in the different layers of the varicose vein wall with the most prominent manifestation in the media. In the third age group of patients with more advanced stage of the disease, higher EC and SMC apoptosis was seen despite lowering the level of Bax and increasing, level of p105/p50 assuming that other signaling cascades may be involved in apoptotic regulation. The impact of the aging process and the progression of inflammation on the NF-kappaB cascade and apoptotic regulation cannot be ruled out. Additional studies with implication of other NF-kappaB subunits and members of Bcl-2 family are essentially needed to draw more substantial conclusions.

## Figures and Tables

**Table 1:** Groups of patients and their characteristics.

Group	Age	No. of patients	CEAP classification*
I	Younger than 35 years (mean age 31 years)	8	C2 (*n* = 6); C3 (*n* = 2)
II	Age between 36–50 years (mean age 42 years)	11	C2 (*n* = 9); C3 (*n* = 2)
III	Older than 50 years (mean age 59 years)	16	C2 (*n* = 12); C4 (*n* = 4)

*CEAP classification: classification system of varicose veins accounting clinical, etiological, anatomical, and pathophysiological aspects.
